# Supplementary material for: Self-Administration of Entactogen Psychostimulants Dysregulates Gamma-Aminobutyric Acid (GABA) and Kappa Opioid Receptor Signaling in the Central Nucleus of the Amygdala of Female Wistar Rats
Source: Front Behav Neurosci. 2021 Dec 16;15:780500. doi: 10.3389/fnbeh.2021.780500 (PMC8716434; doi:10.3389/fnbeh.2021.780500)
Supplement: Supplementary file 1 [file Data_Sheet_1.docx]

**Self-administration of entactogen psychostimulants dysregulates gamma-aminobutyric acid (GABA) and kappa opioid receptor signaling in the central nucleus of the amygdala of female Wistar rats**

***Supplementary Information***

Table of Contents

Supplementary Results 2

Figure S1. Self-administration of Pentylone in All Rats Compared to Rats Selected for Electrophysiological Studies. 2

Figure S2. Self-administration of MDMA in All Rats Compared to Rats Selected for Electrophysiological Studies. 3

Figure S3. Self-administration of Saline in All Rats Compared to Rats Selected for Electrophysiological Studies. 4

Figure S4. Self-administration of MDMA or MDMA-analog in female rats trained to self-administer Pentylone. 5

# Supplementary Results

| 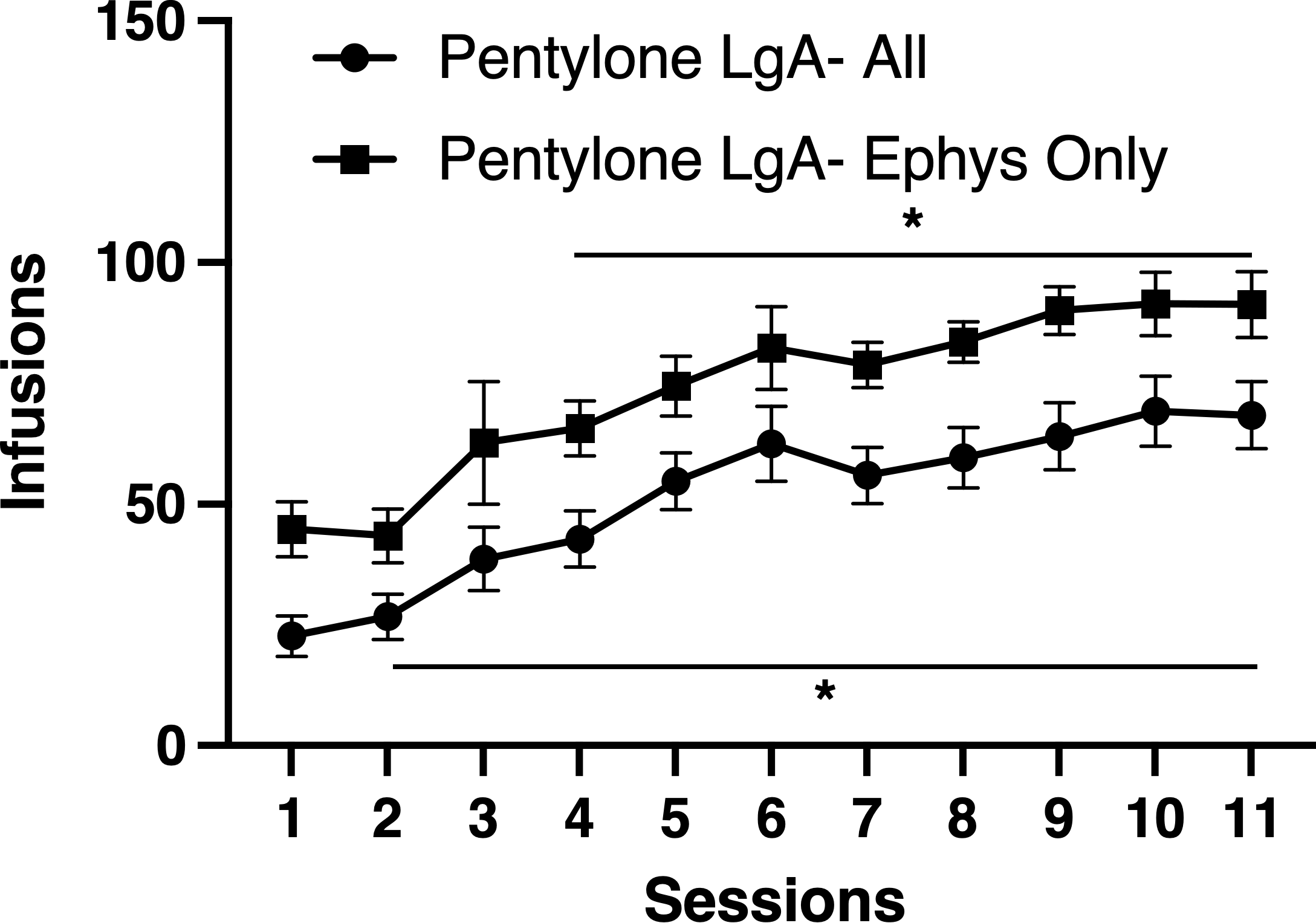 |
| --- |
| Figure S Self-administration of Pentylone in All Rats Compared to Rats Selected for Electrophysiological Studies. *Mean (±SEM) pentylone infusions obtained by long-access rats (Pentylone LgA- All ; N=25) compared to long-access rats selected for ephysiological studies (Pentylone LgA- Ephys Only; N=8) across acquisition training sessions of self-administration. The two-way ANOVA confirmed a significant main effect of Session [F(10,310)=30.42; P<0.0001] and of Group [F(1,31)=4.630; P=0.0393]. Post hoc comparisons confirmed no significant differences (p >0.05). A significant difference from the first session, within group, is indicated with ** |

**
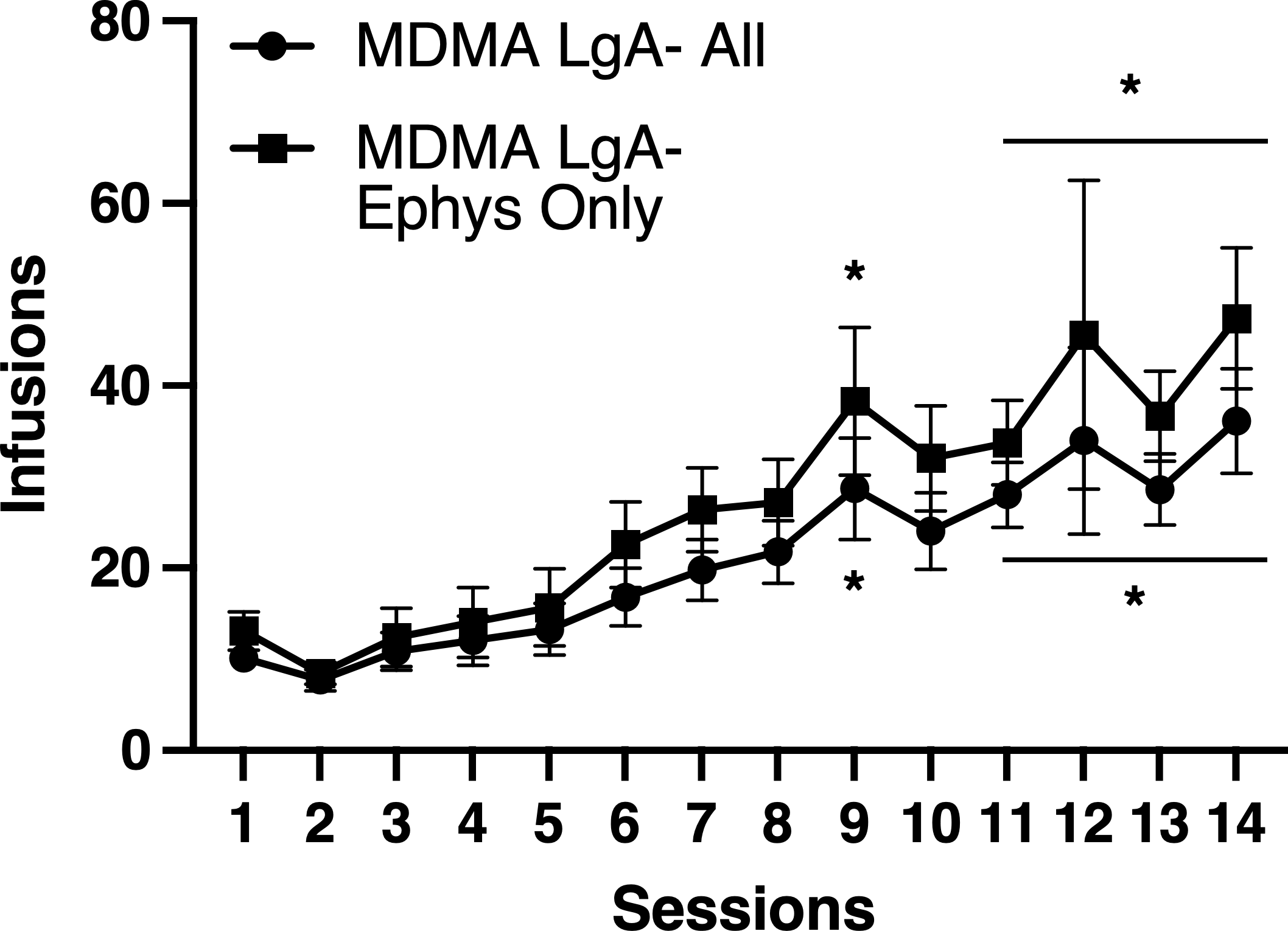
**

## Figure S Self-administration of MDMA in All Rats Compared to Rats Selected for Electrophysiological Studies.

*Mean (±SEM) MDMA infusions obtained by long-access rats (MDMA LgA- All; N=19) compared to long-access rats selected for ephysiological studies (MDMA LgA- Ephys Only; N=11) across acquisition training sessions of self-administration. The two-way ANOVA confirmed a significant main effect of Session [F(13,364)=12.88; P <0.0001] but failed to confirm a significant effect of Group [F(1,28)=1.466; P=0.2361]. A significant difference from the first session, within group, is indicated with **


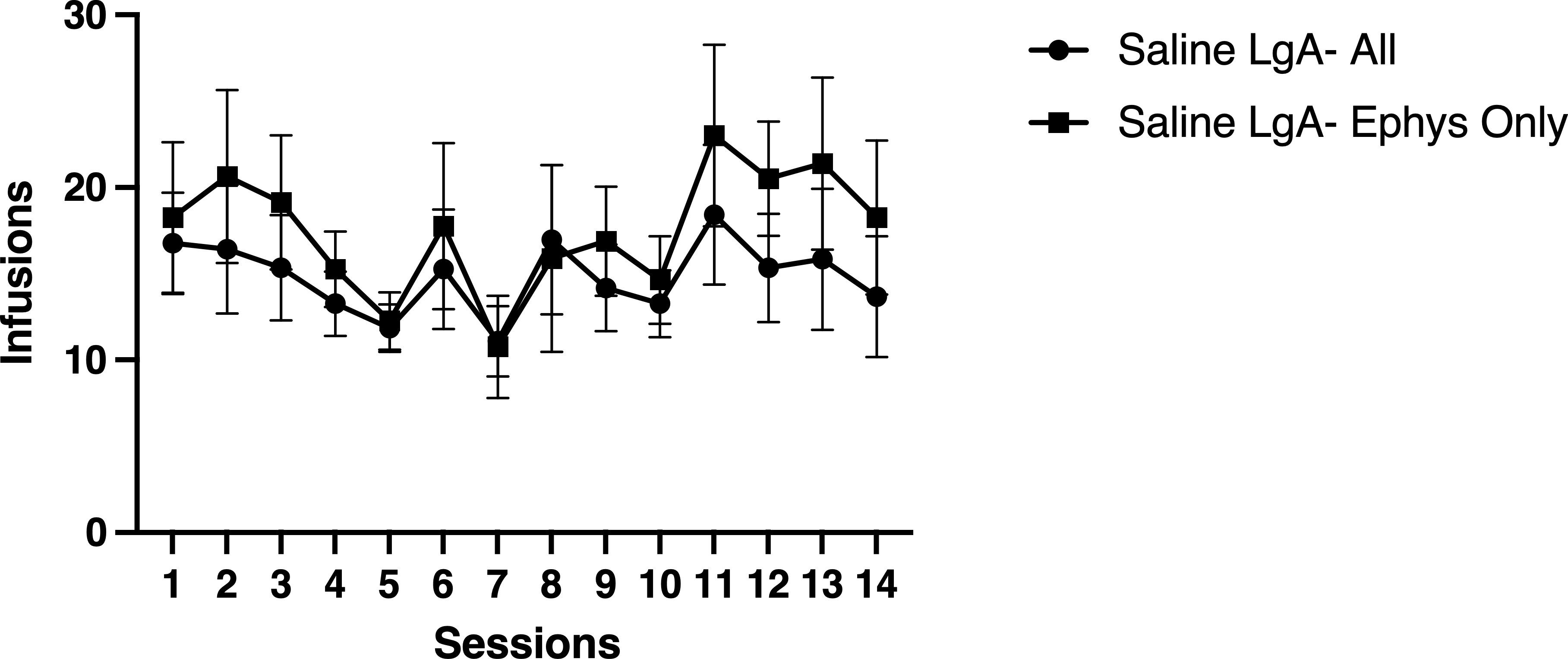


## Figure S Self-administration of Saline in All Rats Compared to Rats Selected for Electrophysiological Studies.

*Mean (±SEM) saline infusions obtained by long-access rats (Saline LgA- All; N=12) compared to long-access rats selected for ephysiological studies (Saline LgA- Ephys Only; N=8) across acquisition training sessions of self-administration. The two-way ANOVA confirmed a significant main effect of Session [F(13,234)=1.987; P=0.0226] but failed to confirm an significant effect of Group [F(1,18)=0.5659; P=0.4616].*


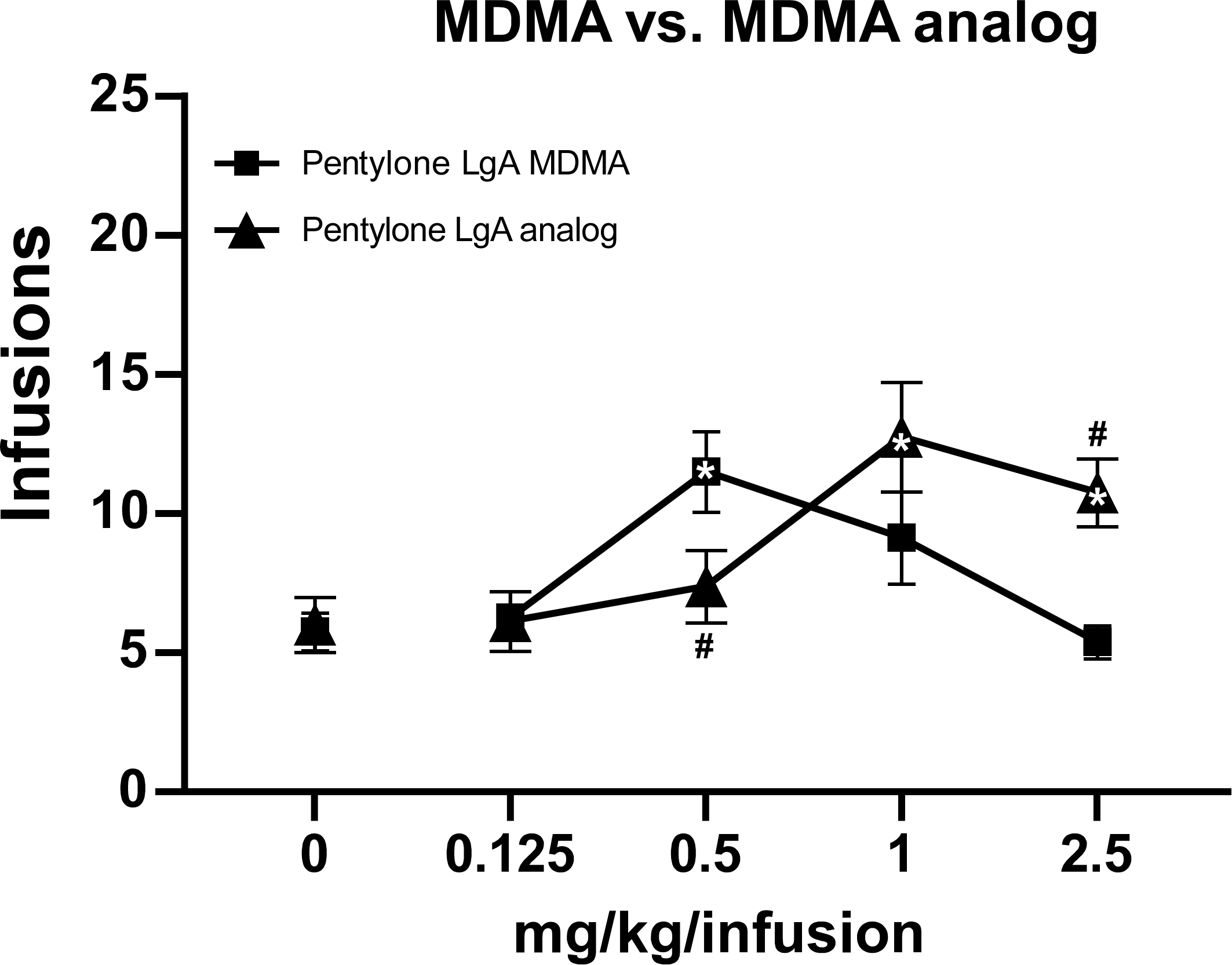


## Figure S Self-administration of MDMA or MDMA-analog in female rats trained to self-administer Pentylone.

*Mean (±SEM) infusions of MDMA or MDMA-analog obtained by Pentylone self-administration trained rats (N=8). Mixed effects analysis confirmed a significant main effect of Dose [F(4,28)=5.857; P = 0.0015] and of the Dose X Group Interaction [F(4,28)=6.603; P = 0.0007]. The post hoc test confirmed the number of MDMA infusions (0.5 mg/kg/infusion) were significantly higher compared to vehicle, whereas the number of MDMA analog infusions was higher at 1 and 2.5 mg/kg/infusion doses. A significant difference from saline, within group, is indicated with * and a significant difference between groups is indicated with #.*
